# Investigation of the removal of diclofop methyl herbicide by peroxy electrocoagulation process and kinetic and cost analysis

**DOI:** 10.1007/s11356-024-33163-7

**Published:** 2024-04-04

**Authors:** Büşra Erden, Gamze Katırcıoğlu Sınmaz, Nazire Pınar Tanattı, Meryem Aksu, İsmail Ayhan Şengil

**Affiliations:** 1 Department of Environmental Engineering, Sakarya University, 54100 Sakarya, Turkey; 2 Department of Environmental Protection Technologies, Sakarya University of Applied Sciences, 54100 Sakarya, Turkey

**Keywords:** Diclofop methyl, Advanced oxidation process, Peroxy electrocoagulation, Fe electrode, Kinetic modelling, Cost analysis

## Abstract

Pesticides containing chlorine, which are released during agricultural activities, are chemical substances that mix with surface and underground waters and have toxic, carcinogenic, and mutagenic effects on the entire living ecosystem. Due to their chemically stable structure, conventional water and wastewater treatment techniques such as coagulation, flocculation, and biological oxidation do not entirely remove these chemical substances. Therefore, before releasing them into the environmental receptor, these chemical substances must be transformed into harmless products or mineralized through advanced oxidation processes. When we look at the literature, there are not many studies on methods of removing diclofop methyl from aquatic media. Our study on the removal of diclofop methyl herbicide from aquatic media using the peroxy electrocoagulation method will provide the first information on this subject in the literature. In addition, this treatment method will contribute significantly to filling an important gap in the literature as an innovative approach for diclofop methyl removal. Moreover, peroxy electrocoagulation, which produces less sludge, provides treatment in a short time, and is economical, has been determined to be an advantageous process. The effects of conductivity, pH, H_2_O_2_ concentration, current, and time parameters on the removal of diclofop methyl were investigated using a GC–MS instrument. Kinetics, energy consumption, and cost calculations were also made. Under the optimum conditions determined (pH = 5, H_2_O_2_ = 500 mg/L, NaCl = 0.75 g/L, current density = 2.66 mA/cm^2^), the peroxydic electrocoagulation process resulted in a diclofop methyl removal efficiency of 79.2% after a 25-min reaction. When the experimental results were analyzed, it was found that the results fitted the pseudo-second-order kinetic model.

## Introduction

In the century we live in, the human population is increasing every day. Agricultural lands are being expanded to meet the nutritional needs of living organisms. In order to increase the production of crops grown in these areas, harmful organisms are being fought against, and pesticides are being used for this purpose. As a result of the unconscious use of pesticides, these agricultural chemicals can spread into the environment and can have a toxic effect on the entire ecosystem directly or indirectly. During application, a portion of the pesticides sprayed evaporates, while the rest penetrates into the plant and soil. The evaporated portion returns to the surface due to air movements and can enter the bodies of non-target organisms, causing residue and toxicity (Akdoğan 2012).

Toxic chlorinated pesticides that cannot be removed by conventional methods should be converted into harmless products by advanced oxidation processes and discharged into receiving environments (Poyatos et al. 2010).

Although advanced oxidation processes have different mechanisms such as photocatalytic and electrochemical reactions, their common goal is to generate the oxidant OH^•^ radicals with the highest electrochemical oxidation potential (2.8 V) after fluoride oxidant (Andreozzi et al. 1999; Li et al. 2022). One of the advanced oxidation processes based on the principle of electrochemical treatment is the peroxy-electrocoagulation process, which is an electrochemical modification of the classic Fenton method that involves both iron ions and hydrogen peroxide. This process is a simple, cost-effective, and environmentally friendly method. Literature shows that this method has been studied for various wastewaters such as detergent wastewater, slaughterhouse wastewater, leachate, olive mill wastewater, aniline-containing wastewater, and synthetic dye-containing textile wastewater (Brillas and Casado 2002; Boysan and Çavunt 2021).

In the conventional Fenton process, both H_2_O_2_ and Fe^+2^ are added externally to the reaction medium. In peroxy electrocoagulation, which is a method that removes pollutants through coagulation, adsorption, precipitation, and flotation mechanisms, H_2_O_2_ is added externally, while Fe^+2^ is produced electrochemically in situ when an iron anode is used, without the need for additional iron to produce Fe^+2^. OH^−^ ions are produced at the cathode. Depending on the setup of the electrolytic cell, Fe^+2^ can be continuously produced at the cathode. Hydroxyl radicals (OH^•^) (Fenton reagents) are formed as a result of the reaction between Fe^+2^ and H_2_O_2_. The peroxy electrocoagulation method is an advantageous method for adsorbing and removing pollutants that need to be removed from the environment, depending on the conditions of the environment, by increasing the concentration of metal-polymer complex structures added to the environment through dissolution from the electrodes over time, and thereby removing small-sized colloidal pollutant particles (Khataee et al. 2009; Boysan and Çavunt 2021).

**Fig. 1:**
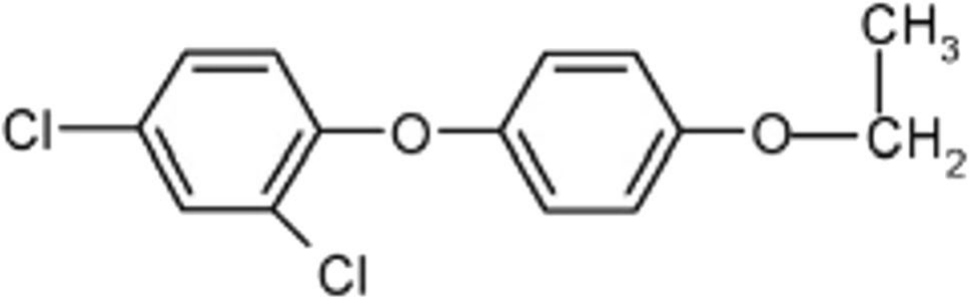
Chemical structure of diclofop methyl (Wu et al. 2020)

Phenoxy herbicides are ranked third among the most commonly used herbicides worldwide (Wu et al. 2020). Diclofop methyl, a type of phenoxy acid herbicide, can also be called Illoxan, Hoelon, Hoe-Gross, and other names. It is derived from aryloxy propionic acid and is a colorless and odorless crystalline structure. The chemical compound has a molecular formula of C1_6_H_14_Cl_2_O_4_, a molecular weight of 341.20, a melting point of 39–41 °C, a boiling point of 173–175 °C under 0.1 mbar pressure, and a vapor pressure of 3.4 × 10^−7^ mbar at 20 °C and 1.5 × 10^−7^ mbar at 30 °C. It is easily soluble in organic solvents such as acetone, xylene, and methanol. For example, it has a solubility of 40 g/100 mL in acetone, 50 g/100 mL in xylene, and 40 g/100 mL in methanol. However, its solubility in water at 220 °C is quite low at 50 mg/L. The chemical structure of diclofop methyl is shown in Fig. 1 (Wu et al. 2020). This pesticide is a post-emergence type and is used to control wild oats and weeds. Its usage areas include the production of crops such as peas, wheat, potatoes, carrots, soybeans, chickpeas, sugar beets, and onions. Due to its persistence and transportability properties, it is heavily used in many countries (Gomathi Devi and Krishnamurthy 2009). This substance is a toxic substance. The degradation byproduct 4-(2,4-dichloro phenoxy) phenol was determined to be more toxic than diclofop methyl (Cai et al. 2009).

When we look at the literature, there are not many studies on the removal methods of diclofop methyl in aquatic environments. The photolytic mineralization of diclofop methyl was investigated using TiO_2_ and BaTiO_3_ as photocatalysts, and complete mineralization was achieved after 2 h, and the degradation mechanism of diclofop methyl was determined (Gomathi Devi and Krishnamurthy 2009). In another study, the removal of two herbicides belonging to the phenoxy acetic acid class, 4-chloro-2-methyl phenoxy acetic acid and 2.4-dichloro phenoxy acetic acid (MCPA and 2,4-D), was successfully performed using commercial activated carbon in aqueous medium (Spaltro et al. 2018). The oxidation of chlorinated phenoxy herbicide 2,4-dichloro phenoxy acetic acid (2,4-D) was carried out in aqueous medium by thermally activated persulfate (Cai et al. 2018). The electro and photoelectrooxidation of 2,4,5-trichloro phenoxiacetic acid (2,4,5-T) was comparatively investigated in an aqueous medium using PbO_2_, Sb-doped SnO_2_, BDD, and TiO_2_-NTs anodes (Santos et al. 2020). The degradation of phenoxy acid and diclofop methyl in aqueous medium was studied with gamma radiation (Zaouak et al. 2020). The toxicity and degradation relationship of diclofop methyl and its two main metabolites, diclofop and 4-(2,4-dichlorophenoxy) phenol, were investigated in three selected freshwater algal cultures (*Chlorella pyrenoidosa*, *Chlorella vulgaris*, and *Scenedesmus obliquus*) (Cai et al. 2007). The photolytic degradation of herbicides (2,4-D (dichloro-phenoxy-acetic acid) and insecticides commonly used in agricultural fields in rinsing water was investigated using titanium-based photocatalysts (Herrmann and Guillard 2000). The degradation and toxicity of diclofop methyl over time were studied in algal suspensions (Cai et al. 2009).

The aim of our study is to investigate the effects of pH, H_2_O_2_ dosage, current density, and time parameters on the treatment efficiency of synthetic wastewater containing 20 mg/L of diclofop methyl pesticide using the peroxy-electrocoagulation process. The study also includes the determination of kinetic models, electrical consumption, and cost calculations at the optimum values. This study presents an effective and innovative purification method for diclofop methyl removal from aquatic media. The peroxide electrocoagulation method, which generates less sludge in the environment compared to other treatment processes, has been applied for diclofop methyl removal. This method enables shorter treatment time, requiring smaller pool volumes. Moreover, its economic efficiency is an additional advantage. In addition, it is aimed to make a significant contribution to the literature on diclofop methyl, one of the chlorinated herbicides.

## Materials and methods

### Chemicals

Diclofop methyl calibration standard was obtained from Sigma-Aldrich (USA) for GC–MS calibration. All chemicals used in the experiments were of analytical purity and were purchased from Merck KGaA (Germany), including acetonitrile (C_2_H_3_N), ethyl acetate (C_4_H_8_O_2_), hydroxide (NaOH), hydrochloric acid (HCl), sodium chloride (NaCl), and hydrogen peroxide (H_2_O_2_, 35%). A synthetic aqueous solution of diclofop methyl was prepared using acetonitrile as the solvent. NaOH and HCl were used to adjust the pH of all solutions, ethyl acetate was used as the organic solvent for extraction studies, and hydrogen peroxide was used as the reactive agent in peroxide electrocoagulation experiments. After the experiments, the plates used as anode and cathode were washed with a dilute HCl solution prepared in a 1:1 volume ratio to remove oxide layers and residues that formed on their surfaces during the reaction process, minimizing time and energy loss. The electrodes were then rinsed with pure water and prepared for the next experiment.

### Instruments

The concentration measurements of diclofop methyl in the experiments were performed using Shimadzu QP 2010 GC/MS device, pH measurements were carried out using Hanna HI 2221 pH meter, and weighing procedures were conducted using And GR-200 precision balance. In addition, Yıldırım Electronics Y-0012 power supply, Heraeus drying oven, MTops HS33 mixer, Merck Millipore Direct-Q 5 UV pure water device, Labtech LVM-202 vacuum pump, and Labtech LVM-202 vortex mixer were used in the experiments.

### Reactor design

**Fig. 2:**
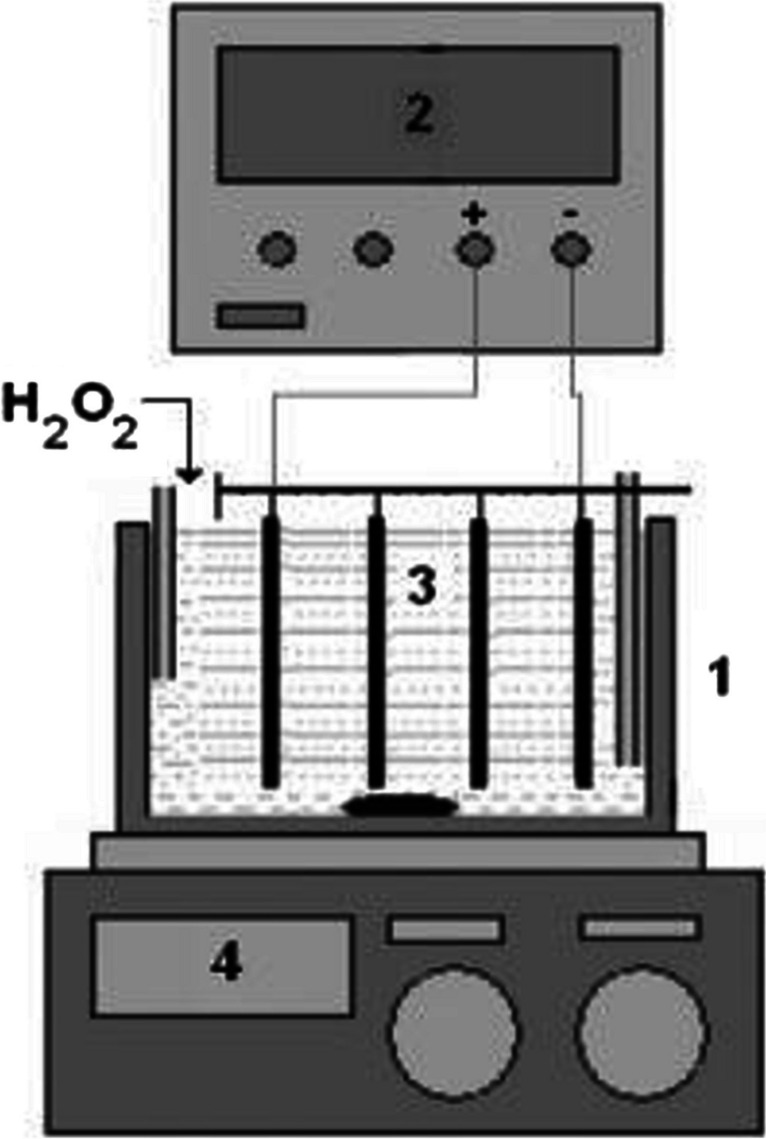
Electrocoagulator (1, batch reactor; 2, dc power supply; 3, bipolar electrodes; 4, magnetic stirrer)

In the peroxy electrocoagulation method, iron electrodes with dimensions of 13 cm × 4.5 cm × 0.2 cm were used as both anode and cathode. Four electrodes were placed in a bipolar configuration within a 250-mL glass beaker, serving as the reactor, with a distance of 1.5 cm between them. The peroxy electrocoagulation reactor setup is given in Fig. 2. The total surface area of the electrodes that came into contact with the solution was 37.6 cm^2^.

In all experiments, diclofop methyl solution at a concentration of 20 mg/L was used in a volume of 100 mL. After four iron electrodes were placed bipolarly in a 250-mL glass beaker, diclofop methyl solution was added into the reactor. After the electrodes were connected to the power source, each experiment was carried out in triplicate at the determined pH, H_2_O_2_ concentration, current density, and reaction time. In all electrochemical experiments, experimentally determined 0.75 g/L sodium chloride was used as the electrolytic material. The stirring speed was set to 200 rpm, and the experiments were performed on a magnetic stirrer. At the end of each experiment carried out under the specified conditions, 3 ml of the sample was taken into 20-ml glass vials and extracted with the same volume of ethyl acetate solution. After adding 3 mL of ethyl acetate to 3 mL of sample, the mixture was vortexed for 1 min and then left for 15 min for phase separation. The supernatant was transferred into 2-ml glass vials and analyzed on the GC–MS device at the specified temperature program. All experiments were performed at room temperature.

### Preparation and measurement of DCM samples

Accurate and simultaneous analyses of phenoxy acid herbicides, including diclofop methyl, can be performed using chromatographic methods such as high-performance liquid chromatography (HPLC), capillary electrophoresis, and gas chromatography (Seebunrueng et al. 2020). The measurements made in our study were carried out with Shimadzu brand QP 2010 GC/MS device ECD detector and Restek brand Rtx-CLPesticides2 (30 m, 0.32 mm ID, 0.25 μm) column in Sakarya University Environmental Engineering Department Water and Wastewater Laboratory. A Sigma-Aldrich brand diclofop methyl calibration standard was used for GC–MS calibration.

The GC–MS temperature program used is as follows: the analysis starts without waiting at 80 °C, and then it is kept for 5 min. After that, the temperature is increased at a rate of 20 °C/min and held at 150 °C for 2 min. Then, it is ramped up to the maximum temperature of 225 °C at a rate of 30 °C/min and kept for 17 min before the program is ended. The injection temperature used in the method is 225 °C, the ion source temperature is 220 °C, and the interface temperature is 175 °C. Helium is used as the carrier gas, and the column pressure is set to 120 kPa, with a total flow rate of 62.8 mL/min. Different versions of these values were tried, but it was decided that the temperature program described above provided the best results.

Solutions in the range of 1–5–10–25–50–75–100 mg/L concentrations were prepared for calibration using a stock solution of 100 mg/L concentration of diclofop methyl with acetonitrile as the solvent. The samples were analyzed in triplicate using the GC–MS instrument with the temperature program specified. A linear calibration curve between 1 and 100 mg/L is shown in Eq. (1). The obtained *R*^2^ value for the calibration curve was 0.999.

1 $$The\;concentration\;of\;diclofop\;methyl \left(\frac{mg}{L}\right)=\frac{Area+1000907}{\mathrm{125947,6}}$$

Before submitting the sample to GC–MS, extraction processes were carried out using ethyl acetate as the solvent (de Pinho et al. 2010). The extraction was performed by adding an equal volume of ethyl acetate to the sample in the vial, followed by vortexing for 1 min and waiting for 15 min for phase separation. After these steps, the upper phase of the solution was taken for measurement by GC–MS using the specified temperature program.

The removal efficiency of diclofop methyl was calculated by the following Eq. (2):

2 $$The\;removal\;efficiency\;of\;diclofop\;methyl \left(\mathrm{\%}\right)= \frac{{C}_{o}-{C}_{t}}{{C}_{0}} x100$$

In Eq. (2), C_o_ is the initial diclofop methyl concentration (mg/L), and C_t_ is the diclofop methyl concentration at time *t* (mg/L).

## Results and discussion

### Effect of conductivity on diclofop methyl removal

**Fig. 3:**
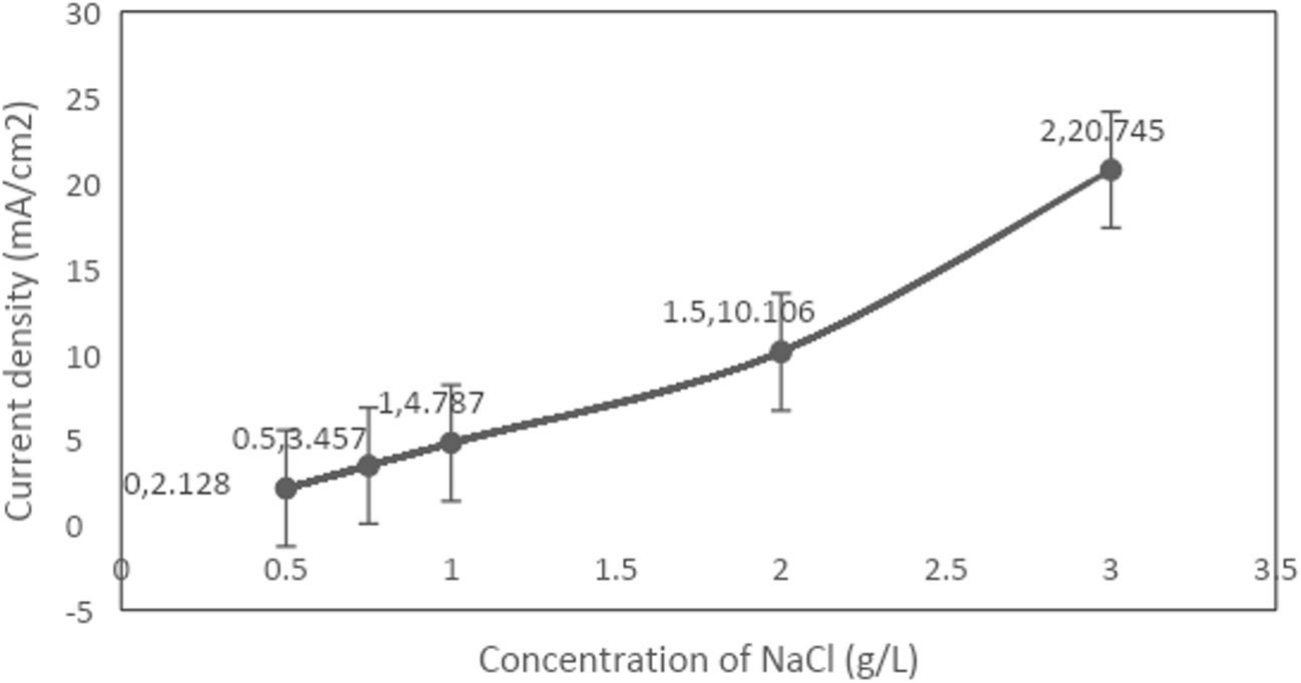
Graph of conductivity-current relationship

Electrolytic addition is necessary for solutions with insufficient conductivity in electrochemical processes. NaCl electrolyte provides the conductivity of the solution and accelerates electron transfer (Jiang and Zhang 2007). A 20-mg/L concentration solution of diclofop methyl was prepared in a volume of 100 mL and transferred to the reactor. Sodium chloride was weighed on a precision balance to concentrations of 0.5, 0.75, 1, 2, and 3 g/L and added to the solution. After the electrodes were placed in the reactor containing the solution and connected to the power supply, the control knobs that adjust the voltage and current of the power supply were set to the maximum level, and the current values achieved with different doses of sodium chloride were determined. The results are given in Fig. 3.

As seen from the graph, the sodium chloride concentration that provides the conductivity for the maximum planned current density value of 3.19 mA/cm^2^ is 0.75 g/L. Therefore, it was decided to use this dose of sodium chloride in the electrochemical experiments to be carried out in order to prevent additional electrolytic substances and extra costs.

### Effect of pH on diclofop methyl removal

**Fig. 4:**
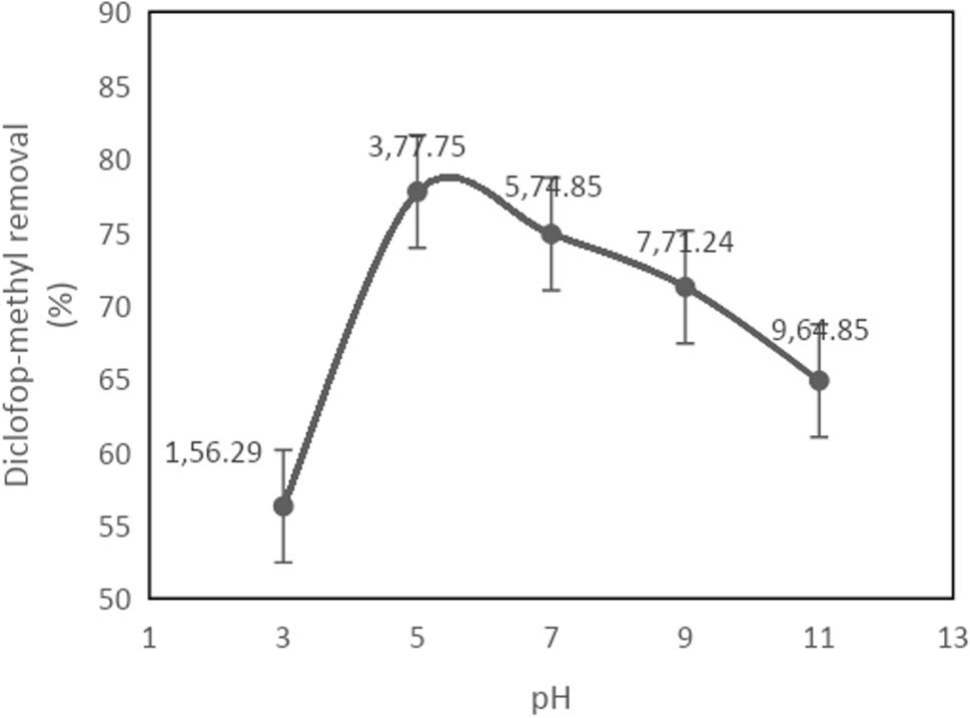
The effect of pH on diclofop methyl removal in the peroxy electrocoagulation process (C_0_ = 20 mg/L, H_2_O_2_ = 500 mg/L, current density = 2.66 mA/cm.^2^, NaCl = 0.75 g/L, *t* = 5 min)

Synthetic wastewater samples containing diclofop methyl at a concentration of 20 mg/L in 100 mL were prepared separately with pH values of 3, 5, 7, 9, and 11. The process parameters were kept constant with a hydrogen peroxide concentration of 500 mg/L, a sodium chloride concentration of 0.75 g/L, and a current density value of 2.66 mA/cm^2^. Electrodes were placed in the 250-mL glass reactor and connected to the power source. The mixing speed was set at 200 rpm, and experiments were conducted on a magnetic stirrer. After 5 min, the power supply was turned off, and a 3-mL sample was drawn from the reactor with an automatic pipette and transferred to 20-mL glass vials for extraction with ethyl acetate. After adding 3 mL of ethyl acetate to the 3-mL sample, the mixture was vortexed for 1 min and then allowed to stand for 15 min for phase separation. The upper phase was drawn off with an automatic pipette and transferred to 2-mL glass vials. The analysis was performed with a GC–MS device using the temperature program determined. Each experiment was conducted in triplicate, and the diclofop methyl removal percentages were calculated by taking the average. All experiments were conducted at room temperature. The experiment results showing the effect of pH values on diclofop methyl removal are presented in Fig. 4.

It has been reported in the literature that pH is one of the most important parameters affecting the efficiency of electrocoagulation processes for the removal of organic pollutants. In a medium where the pH is between 2 and 5 in the presence of Fe^2+^ ions and organic pollutants, the catalytic decomposition of H_2_O_2_ leads to the formation of OH^•^ radicals (Boysan and Çavunt 2021). At low pH values, H_2_O_2_ causes the deactivation of iron by providing a stable complex formation of iron species. At high pH values, lower removal efficiency is due to the instability of H_2_O_2_ and the formation of iron-III-hydroxide complexes. This leads to a decrease in the production of hydroxyl radicals in the solution and hence a decrease in removal efficiency (Govindan et al. 2014; Venu et al. 2014). Fe(OH)_3_ will precipitate at high pH values, leading to the decomposition of H_2_O_2_ to H_2_O and O_2_ (Şengil and Özacar 2006). It has been observed that acidic pH is more suitable for diclofop methyl removal than neutral and alkaline pHs (Govindan et al. 2014). In a study examining the removal of pesticide (metribuzin) concentration by electrochemical method, it was stated that the concentration decrease was highest in the pH range of 5 to 6 (Yahiaoui et al. 2011). As shown in Fig. 3, the highest removal efficiency is achieved at pH 5. In the experiments examining the parameters affecting the process, the pH will be kept constant at 5.

### Effect of H_2_O_2_ on diclofop methyl removal

**Fig. 5:**
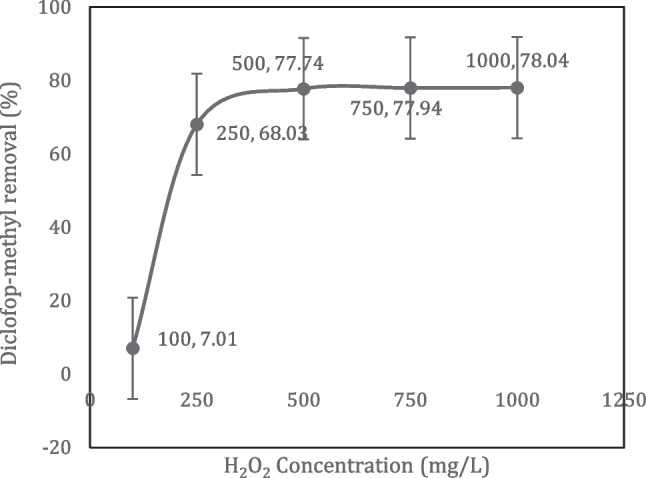
The effect of H_2_O_2_ dosage on the removal of diclofop methyl using the peroxy electrocoagulation process (pH = 5, C_0_ = 20 mg/L, current density = 2.66 mA/cm.^2^, NaCl = 0.75 g/L, *t* = 5 min)

In the study of the effects of H_2_O_2_ concentration on the removal of diclofop methyl, pH 5, initial diclofop methyl concentration of 20 mg/L, current density of 2.66 mA/cm^2^, NaCl concentration of 0.75 g/L, and stirring speed of 200 rpm was kept constant. Experiments were conducted separately by adding 100, 250, 500, 750, and 1000 mg/L H_2_O_2_ to the 100-mL-volume samples taken in the glass reactor. After the electrode arrangement was placed and the iron electrodes were connected to the power supply adjusted to 2.66 mA/cm^2^, the reaction was started on the stirrer and stopped by turning off the power supply after 5 min. The sample taken in a volume of 3 mL was extracted with an equal volume of ethyl acetate. After 1 min of mixing and 15 min of waiting steps in the vortex device, the sample was analyzed with the GC–MS device. The experiments were performed in triplicate at room temperature. The experimental results on the effects of H_2_O_2_ concentration on the removal of diclofop methyl are presented in Fig. 5.

As seen in Fig. 4, the removal of diclofop methyl has increased with the addition of H_2_O_2_ up to a specific dose. The addition of H_2_O_2_ leads to the formation of more hydroxyl radicals in the process and oxidizes organic matter that cannot be adsorbed by coagulants (Bashir et al. 2019). There was no significant change in the removal efficiency after the addition of 500 mg/L H_2_O_2_. The reason for this is that if more H_2_O_2_ is added than the optimum dose determined for the system, H_2_O_2_ reacts with hydroxyl radicals, reducing the hydroxyl radicals in the medium and producing hydroperoxyl radicals (E° = 1.70 V) which are less reactive than hydroxyl radicals (E° = 2.80 V) (Freitas et al. 2014). Therefore, the removal efficiency does not increase (Ghanbari and Moradi 2015). Since no significant change was observed in the diclofop methyl removal efficiency after the addition of 500 mg/L H_2_O_2_ dose, it was decided to use 500 mg/L H_2_O_2_ as the optimum H_2_O_2_ dose in the experiments.

### Effect of current density on diclofop methyl removal

**Fig. 6:**
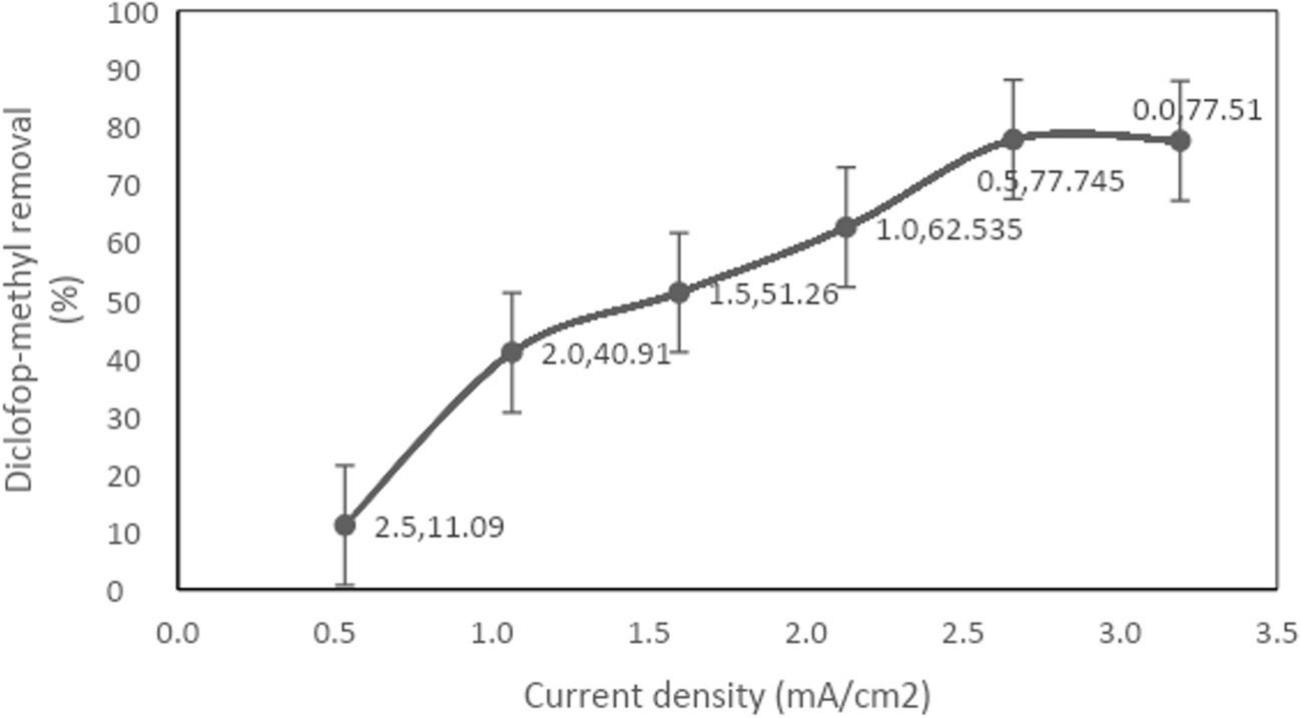
The effect of current density on diclofop methyl removal in the peroxy electrocoagulation process (pH = 5, C_0_ = 20 mg/L, H_2_O_2_ = 500 mg/L, NaCl = 0.75 g/L, *t* = 5 min)

During the investigation of the effects of current intensity on the removal of diclofop methyl, the pH 5, initial diclofop methyl concentration of 20 mg/L, H_2_O_2_ concentration of 500 mg/L, NaCl concentration of 0.75 g/L, and stirring speed of 200 rpm were kept constant. A 100-mL volume of 20 mg/L diclofop methyl solution was added to a glass reactor, and the reaction was initiated by turning on the power supply, which was set to a constant value of 0.02 A, and stirring on the stirrer. After 5 min, the power supply was turned off, the reaction was stopped, and a sample was taken and transferred to the extraction stage. The sample, which was taken in a volume of 3 mL, was extracted with an equal volume of ethyl acetate. After mixing for 1 min in a vortex device and waiting for 15 min, the sample from the upper phase was taken for analysis on a GC–MS device. The same procedure was repeated for current values of 0.02, 0.04, 0.06, 0.08, 0.1, and 0.12 A. The current density was obtained by dividing the applied current by the total surface area of the electrodes, which was 37.6 cm^2^. The current densities for the determined amperage values were 3.191, 2.660, 2.128, 1.596, 1.064, and 0.532 mA/cm^2^, respectively. The experiments were carried out in triplicate at room temperature. The results of the experiments, which investigated the effects of current intensity on the removal of diclofop methyl, are presented in Fig. 6.

As seen in Fig. 5, the removal efficiency of diclofop methyl increases as the current density increases and then reaches a constant value. This is due to the increase in the production rates of H_2_O_2_ and hydroxyl radicals as the applied current increases. At high current values, the anodic dissolution of iron increases, resulting in the formation of more Fe^+2^ and Fe(OH)_n_(solid) (Yüksel 2009; Yüksel et al. 2009). As the current increases, the electrolytic dissolution rate at the Fe anode increases; subsequently, monomeric Fe(OH)_3_ and polymeric Fe(OH)_n_ flocs are formed, and these insoluble flocs ensure the removal of diclofop methyl (Govindan et al. 2015). Applying more current density than necessary increases the cost and may cause an increase in the amount of sludge in the system. Therefore, in our experimental study, the optimum current density was selected as 2.66 mA/cm^2^.

### Effect of reaction time on diclofop methyl removal

**Fig. 7:**
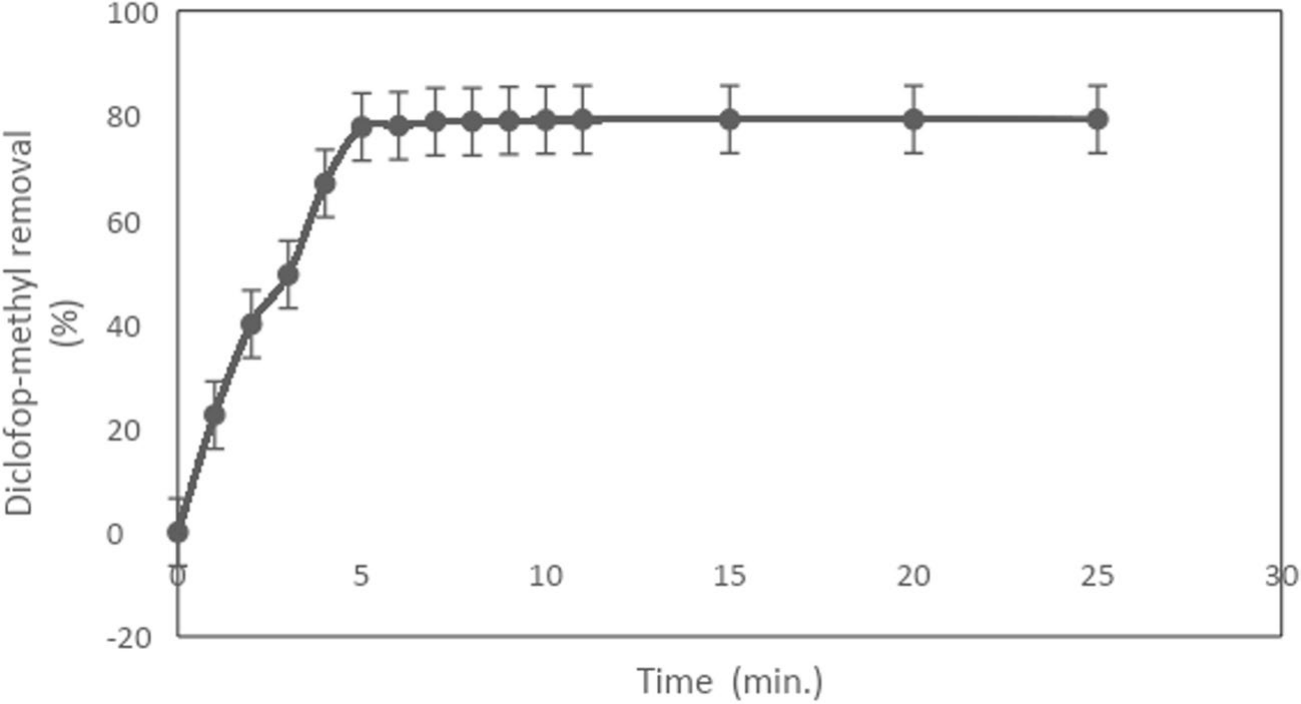
The effect of time on diclofop methyl removal in the peroxy electrocoagulation process (C_0_ = 20 mg/L, pH = 5, H_2_O_2_ = 500 mg/L, NaCl = 0.75 g/L, current density = 2.66 mA/cm.^2^)

After deciding on the optimum pH, H_2_O_2_ amount, and current density values, the percentages of diclofop methyl removal were investigated over time. The values of pH 5, initial diclofop methyl concentration of 20 mg/L, H_2_O_2_ amount of 500 mg/L, current density of 2.66 mA/cm^2^, NaCl concentration of 0.75 g/L, and stirring speed of 200 rpm were kept constant. A 100-mL volume of 20 mg/L diclofop methyl solution was taken into a glass reactor, and the reaction was started on the stirrer by placing the electrode arrangement and running the power supply. The current density was 2.66 mA/cm^2^. At 1, 2, 3, 4, 5, 6, 7, 8, 9, 10, 11, 15, 20, and 25 min, a 3-mL volume of sample was taken, and a 1:1 volume of ethyl acetate was added to the sample, which was mixed for 1 min using a vortex device. After 15 min, the sample in the upper phase was taken and analyzed using a GC–MS device. The experiments were conducted in triplicate at room temperature. The experimental results on the effects of time on diclofop methyl removal are presented in Fig. 7.

The lifetime of the hydroxyl radical is only a few nanoseconds, so it is very short and can only react where it is formed. In the early stages of the reaction, where the organic pollutant is present in high concentrations, the probability of collision between the pollutant molecules and hydroxyl radicals is very high, which leads to an increase in the removal percentage. After a certain point, the removal rate slows down and becomes constant (Venu et al. 2014). As shown in Fig. 7, the removal rate is fast in the first 5 min and then stabilizes.

### Kinetic analysis of diclofop methyl removal efficiencies

The removal of diclofop methyl using the peroxy electrocoagulation process was investigated, and first-order, second-order, pseudo-first-order, and pseudo-second-order kinetic models were calculated to determine which kinetic model best fits the oxidation kinetics.

The first-order equation is given in Eq. (3), and the second-order equation is given in Eq. (4). C_0_ represents the initial concentration of diclofop methyl (mg/L), *C* is the concentration of diclofop methyl removed at time *t* (mg/L), *k* is the oxidation rate constant (1/min), and *t* is the time (min). The pseudo-first-order equation is given in Eq. (5), and the pseudo-second-order equation is given in Eq. (6). Here, *C* represents the remaining concentration of diclofop methyl in the aqueous phase at time *t* (mg/L), Ce is the concentration coefficient (mg/L), and *k*_1_ and *k*_2_ are the oxidation rate constants (L/mg.min) (Tanattı, 2015).

3 $$ln \left(\frac{{C}_{0}}{C}\right)=k.t$$

4 $$\frac{1}{C}-\frac{1}{{C}_{0}}=k.t$$

5 $$log \left({C}_{e}-C\right)=log\;{C}_{e} - \frac{{k}_{1}}{\mathrm{2,303}} t$$

6 $$\frac{t}{C}=\frac{1}{{k}_{2} {C}_{e}^{2}}+ \frac{1}{{C}_{e}} t$$

In Table 1, *R*^2^ and *k* values calculated for four different kinetic models of the peroxy electrocoagulation process are given.

**Table 1:** *R*^2^ and *k* values of kinetic models

Kinetic model	*R*^2^	*k*
First-order	0.9157	0.1533
Second-order	0.796	0.0128
Pseudo-first-order	0.9282	0.4847
Pseudo-second-order	0.9882	0.0432

When Table 1 was examined, the second-order kinetic model with a regression coefficient of 0.796 was found to be the most insignificant model. The regression coefficients of the first-order and pseudo-first-order kinetic models were found to be 0.9157 and 0.9282, respectively. A significance level was found between them within a 10% confidence interval. However, the regression coefficient of the pseudo-second-order kinetic model is 0.9882. Among these four models, the pseudo-second-order kinetic model was determined to be the most significant. In addition, the reaction rate constant was found to be 0.0432 in the pseudo-second-order kinetic model.

**Fig. 8:**
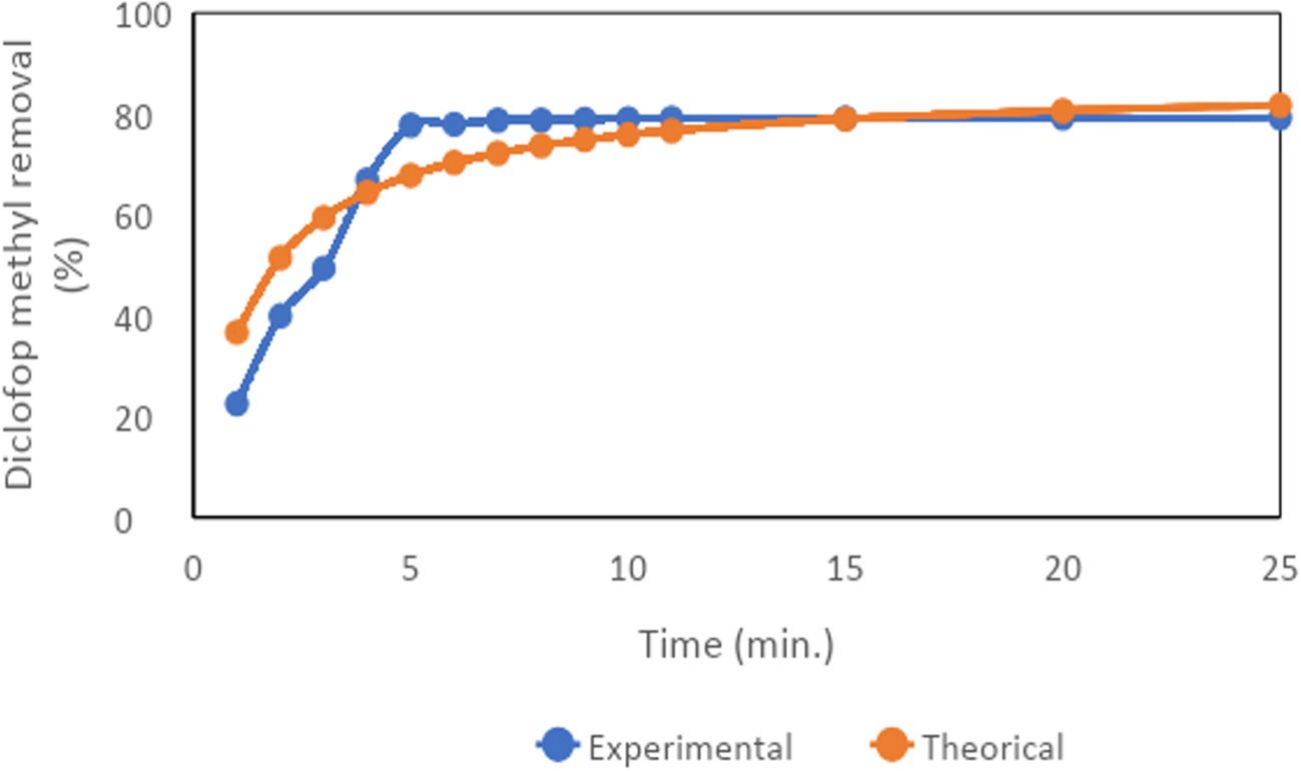
The experimental and theoretical values of the removal efficiency of diclofop methyl calculated using the pseudo-second-order model

Theoretical diclofop methyl removal efficiencies were calculated using the pseudo-second-order kinetic model in the removal of diclofop methyl by the peroxy electrocoagulation method. Theoretical and experimental diclofop methyl removal efficiencies are given in Fig. 8. When Fig. 8 is examined, a rapid diclofop methyl removal occurs theoretically and experimentally in the first 5 min. When experimental and theoretical results are compared, both results overlap in longer time periods (for example, after the tenth minute).

### Energy and cost analysis

The values consumed per cubic meter of wastewater for the parameters affecting the cost in the peroxy electrocoagulation process, which are electric energy, H_2_O_2_ quantity, NaCl quantity, and HCl quantity, have been calculated. The equation given in Eq. (7) has been used to calculate the consumption of electric energy (EEC). The total cost calculation is presented in Table 2.

**Table 2:** Cost analysis of diclofop methyl wastewater treatment using the peroxy electrocoagulation process

Parameter	Consumption	Unit price ($)	Cost ($)
Electric energy	10.125 kWh/m^3^	0.068	0.688
H_2_O_2_	1.260 L/m^3^	29.29	36.9
NaCl	0.75 kg/m^3^	11.75	8.813
HCl	0.2 L/m^3^	26.2	5.24
		**Total cost**	51.641 $/m^3^

7 $$EEC=\frac{{\int }U I dt}{\left({C}_{0}-{C}_{t}\right) V 3.6}=\frac{I{\int }U dt}{\left({C}_{0}-{C}_{t}\right) V 3.6}$$

In Eq. (7), *U* is the applied voltage (V), *I* represents the current value (A), *t* is the electrolysis time (min), C_o_ is the initial diclofop methyl concentration (mg/L), C_t_ is the diclofop methyl concentration at time *t* (mg/L), and the value of *V* is the wastewater volume (L) (Boysan and Çavunt 2021).

It has been concluded that the peroxy electrocoagulation process, which achieved 79.2% removal of diclofop methyl after a 25-min reaction, with a total cost of 51.641 $/m^3^, is both effective and economical.

## Conclusions

Diclofop methyl, one of the phenoxy acid herbicides, is extensively used globally to control the growth of weeds for improving and enhancing agricultural production. These herbicides are classified as moderately toxic by the WHO. Due to their high polarity, these herbicides pass from agricultural fields to surface and groundwater, negatively impacting the ecosystem (Cao et al. 2020; Seebunrueng et al. 2020).

There is limited research on the removal of diclofop methyl from water in the literature. This study was conducted to contribute to the literature by investigating the removal of diclofop methyl from water using the peroxy electrocoagulation process and examining the effects of conductivity, pH, H_2_O_2_ dosage, current density, and time parameters. Synthetic wastewater containing 20 mg/L of diclofop methyl was prepared in the laboratory, and measurements were taken using a GC–MS device according to the temperature program specified for this substance.

Experimental studies conducted on the removal of diclofop methyl by peroxy electrocoagulation process have reached a removal rate of 79.2% after a 25-min reaction time with the optimum values of pH = 5, H_2_O_2_ = 500 mg/L, current density = 2.66 mA/cm^2^, and NaCl = 0.75 g/L.

When the kinetic model calculations were made for the studied oxidation process, it was concluded that the oxidation kinetics fit the pseudo-second-order-kinetic model. The *R*^2^ value was calculated as 0.9882, and the *k* value as 0.043166.

According to the electricity consumption and cost calculation study, 79.2% removal of diclofop methyl was achieved by the peroxy electrocoagulation process in a 25-min reaction, with an energy consumption of 10.125 kWh/m^3^ and a total cost of 51.641 $/m^3^. Considering the amount of diclofop methyl removed and the cost values, it was concluded that the peroxy electrocoagulation process is an economically feasible process.

## Data Availability

All the results are original; the availability of data and materials has not been used in this manuscript.
